# Highly Invasive *Listeria monocytogenes* Strains Have Growth and Invasion Advantages in Strain Competition

**DOI:** 10.1371/journal.pone.0141617

**Published:** 2015-11-03

**Authors:** Evangelia A. Zilelidou, Kathrin Rychli, Evanthia Manthou, Luminita Ciolacu, Martin Wagner, Panagiotis N. Skandamis

**Affiliations:** 1 Laboratory of Food Quality Control and Hygiene, Department of Food Science and Human Nutrition, Agricultural University of Athens, Athens, Greece; 2 Institute for Milk Hygiene, University of Veterinary Medicine Vienna, Vienna, Austria; 3 “Dunarea de Jos” University of Galaţi, Galaţi, Romania; University of Illinois at Chicago College of Medicine, UNITED STATES

## Abstract

Multiple *Listeria monocytogenes* strains can be present in the same food sample; moreover, infection with more than one *L*. *monocytogenes* strain can also occur. In this study we investigated the impact of strain competition on the growth and *in vitro* virulence potential of *L*. *monocytogenes*. We identified two strong competitor strains, whose growth was not (or only slightly) influenced by the presence of other strains and two weak competitor strains, which were outcompeted by other strains. Cell contact was essential for growth inhibition. *In vitro* virulence assays using human intestinal epithelial Caco2 cells showed a correlation between the invasion efficiency and growth inhibition: the strong growth competitor strains showed high invasiveness. Moreover, invasion efficiency of the highly invasive strain was further increased in certain combinations by the presence of a low invasive strain. In all tested combinations, the less invasive strain was outcompeted by the higher invasive strain. Studying the effect of cell contact on *in vitro* virulence competition revealed a complex pattern in which the observed effects depended only partially on cell-contact suggesting that competition occurs at two different levels: i) during co-cultivation prior to infection, which might influence the expression of virulence factors, and ii) during infection, when bacterial cells compete for the host cell. In conclusion, we show that growth of *L*. *monocytogenes* can be inhibited by strains of the same species leading potentially to biased recovery during enrichment procedures. Furthermore, the presence of more than one *L*. *monocytogenes* strain in food can lead to increased infection rates due to synergistic effects on the virulence potential.

## Introduction

Bacteria socialize. Social acts of microbes range from competitive and “microbe-kill-microbe” interactions to cooperative and remarkable self-sacrifice behaviors [1,2]. Competition as a form of microbial interaction involves different types of mechanisms that bacterial cells deploy against potential antagonists. Quorum sensing entails a population-dependent production of signaling molecules, while the contact-dependent- growth inhibition system (CDI) mediates, through cell-contact, the delivery of toxic compounds to bacterial cells in close proximity. Both systems support the survival and growth of one specific strain or species in a complex microbial environment [3–9].

Microbial competition is also critical for survival and proliferation in food products and in food related environments. Foods harbour a great variety of diverse bacterial species and strains, which require common nutritional resources and thus compete for the same niche [1,10–13]. Furthermore, since food products can serve as carriers for pathogenic bacteria, the role of competitive interactions between pathogens and native food microbiota has received considerable attention [14–17]. The ability of pathogenic microorganisms to survive and grow in foods depends not only on the structural characteristics and chemical composition of the food matrix but also on the dynamics of microbial communities present there [18,19].

*Listeria monocytogenes* is a gram-positive, food-borne pathogen, able to switch from a saprophytic life-style to an invasive, intracellular bacterium [20]. It is the causative agent of the rare but severe infectious disease listeriosis. The ubiquitous nature of *L*. *monocytogenes* along with its ability to survive in adverse environments (e.g. low temperatures, low pH) makes this bacterium a major food-safety concern [21].

Different types of interactions between *L*. *monocytogenes* and other food-related bacteria have been investigated. It has been shown that various bacterial species such as members of the lactic acid bacteria, display antimicrobial activity against *L*. *monocytogenes* [15,17,22,23]. In addition, the competitive microbiota of food is known to have a significant effect on the detection of *L*. *monocytogenes* during the enrichment process [24]. *L*. *monocytogenes* faces competition not only from different bacterial species but also from other *Listeria* spp. [25,26]. For example, *L*. *innocua* has been identified as a potential antagonist of *L*. *monocytogenes* able to suppress the growth and to reduce its detectability during enrichment procedures [25–29].

While competition of *L*. *monocytogenes* with other bacteria including other *Listeria* species has been described, little is known about *L*. *monocytogenes* inter-strain interactions. Only two recent studies have demonstrated different recovery rates of *L*. *monocytogenes* strains during the selective enrichment process, as a result of strain competition [30,31]. In contrast, Pan et al. reported no effect of strain competition on biofilm formation of *L*. *monocytogenes* [32].

Whether strain competition affects the growth and *in vitro* virulence of *L*. *monocytogenes* is still unknown. Therefore, we investigated the impact of co-culture on i) growth of *L*. *monocytogenes* strains in nutrient-rich broth and ii) invasion and intracellular proliferation of *L*. *monocytogenes* strains using human intestinal epithelial Caco2 cells. Our hypothesis is that *L*. *monocytogenes* strains that are strong competitors during growth might also have a competitive advantage in their invasion and intracellular growth potential. Furthermore, we investigated whether the observed growth and *in vitro* virulence competition is dependent on cell-contact.

## Materials and Methods

### Bacterial strains

The *L*. *monocytogenes* strains used in this study are listed in Table 1. The strain selection was based on the following criteria: Strain ScottA was selected as a reference human isolate, known to be virulent. The persistent strain 6179 was selected due to its harbors a truncated *internalin A* (*inlA*) resulting in attenuated invasion in Caco2. Strains C5 showed high recovery rate during the enrichment process, whereas strain PL25 revealed only a modest recovery rate after enrichment (unpublished data). Furthermore, to exclude the influence of the individual growth rates on growth competition we selected strains showing a similar growth rate when grown singly.

Strains were characterized by multiplex serogroup-specific PCR according to Doumith et al. [33] and Multilocus Sequence Typing according the Institute Pasteur website (http://www.pasteur.fr/recherche/genopole/PF8/mlst/Lmono.html).

**Table 1:** *L*. *monocytogenes* strains used in this study.

Strain	Antibiotic resistance	Serotype^a^	MLST	Source	Year of isolation	Country
ScottA	Streptomycin Rifampicin	4b	ST290	human isolate	1983	USA
C5	Streptomycin	4b (4d, 4e)	ST2	cow feaces	2007	Ireland
PL25	Rifampicin	1/2b (3b, 7)	ST59	ground pork	2009	Greece
6179	Rifampicin	1/2a (3a)	ST121	cheese	1999	Ireland

^a^ Serovar-specific groups were determined by multiplex PCR. Serotypes in parenthesis were excluded due to MLST classification.

Artificial antibiotic resistance to rifampicin (AppliChem) or streptomycin (Streptomycin Sulfate Biochemica, AppliChem) was induced to the strains for selective enumeration purpose according to Blackburn et al. [34] resulting in higher minimal inhibitory concentrations (MIC) for resistant strains compared to parental strains (S1 Table).

Strains were grown on tryptic soy agar (LABM LB004) supplemented with 0.6% yeast extract (LABM MC001, TSA-Y, sensitive strains) and TSA-Y containing rifampicin (50 μg/ml) or streptomycin (1000 μg/ml) for resistant strains (Rif^R^ and Str^R^). Strains were stored at -80°C, in tryptic soy broth (LABM LB004) containing 0.6% yeast extract (TSB-Y, pH 7.2) supplemented with 20% glycerol.

To ensure that *L*. *monocytogenes* strains did not acquire cross-resistance during the experiments we plated the strains prior each experiment on two selective agars (TSB-Y containing streptomycin or rifampicin) and non-selective TSB-Y agar. Furthermore, after the respective experiments bacteria were not only plated on TSB-Y agar containing rifampicin or streptomycin, but also on non-selective TSB-Y agar. The number of bacteria on TSB-Y agar was equal than the sum of bacteria recovered from both selective agars.

### Growth experiments

One singe colony was inoculated into 10 ml TSB-Y supplemented with either rifampicin (50 μg/ml) or streptomycin (1000 μg/ml) and incubated for 24 h at 30°C. Subsequently 100 μl of this culture were transferred to 10 ml TSB-Y supplemented with the corresponding antibiotic and incubated for 18 h at 30°C. The bacterial cultures (corresponding to approx. 10^9^ CFU/ml) were washed twice with Ringer solution (LABM, LAB100Z) and resuspended in 10 ml of TSB-Y. Subsequently, the cultures were serially diluted in TSB-Y to obtain a final inoculum of approximately 10^3^ CFU/ml. Strains were grown at 10°C for 10 days as individual cultures or in combinations by mixing a rifampicin resistant strain with a streptomycin resistant strain (ratio 1:1, final volume 10 ml).

Cultures were sampled on day 0, 1, 3, 5, 7 and 10; and CFUs were determined by plating serial dilutions on TSA-Y or TSA-Y supplemented with rifampicin or streptomycin. Each experiment was independently performed three times in duplicate.

### *In vitro* virulence assay

Human intestinal epithelial Caco2 cells (ATCC^®^ HTB-37^TM^) were grown in Eagle’s minimum essential medium (MEM), supplemented with 10% (v/v) fetal calf serum (FCS), 2 mM L-Glutamine, 100 units/ml Penicillin, 100 μg/ml Streptomycin sulfate, 0.25 mg/ml Amphotericin and 1% (v/v) non-essential amino acids (all from PAA), at 37°C in a humidified atmosphere (95% relative humidity) containing 5% CO_2_.

Invasion efficiency and intracellular proliferation were determined as previously described by Pricope-Ciolacu et al. [35]. Briefly, Caco-2 cells were seeded into 24-well tissue culture plates and incubated in MEM without antibiotics and containing 0.1% (v/v) bovine serum albumin (BSA; PAA) 24 h prior the experiments.

Bacterial cells were cultivated similar to the growth experiments at 10°C for 24 h except for the higher inoculum level (10^6^ CFU/ml) and the different culture volume (30ml TSB-Y in 50ml plastic tubes). At 24 h, no differences in the populations between the single and mixed strain cultures were observed suggesting no effect of different inoculation levels on *in vitro* virulence. Bacterial cultures were centrifuged (18.0 x g for 5 min at 10°C) and resuspended in MEM (pre-warmed at 37°C) to obtain a multiplicity of infection of 25. Confluent cell monolayers were infected with the cultures for 1 h at 37°C. The cells were washed twice with Dulbecco’s Phosphate Buffered Saline (DPBS) and incubated in MEM containing 0.1% BSA and 100 μg/ml gentamicin (PAA), either for 45 min (invasion assay) or 4 h (intracellular proliferation assay). Subsequently the infected Caco-2 cells were washed twice with DPBS and the intracellular *L*. *monocytogenes* cells were harvested by lysing the Caco-2 cells with 1 ml of cold 0.1% Triton X-100 (Merck, Darmstadt, Germany). The numbers of viable *L*. *monocytogenes* cells after 45 min or 4h of incubation were determined by plating appropriate serial dilutions on TSA-Y and TSA-Y supplemented with rifampicin or streptomycin. CFU were counted after 2 days of incubation at 37°C. Invasion efficiency was calculated as the percentage of initial inoculum recovered by enumeration of intracellular *L*. *monocytogenes* after invasion assay. The intracellular growth coefficient (IGC) was calculated as followed:

$$IGC=\frac{intracellular\;bacteria\;after\;4h-intracellular\;bacteria\;after\;45\;minutes}{intracellular\;bacteria\;after\;45\;minutes}$$

All experiments were performed in triplicate at least three independent times.

### Contact-dependent co-cultivation and *in vitro* virulence experiments

Bacterial cultures were inoculated in TSB-Y as described for growth experiments. Polyethylene tetraphthalate (PET) track-etched membrane inserts of 0.4 μm pore size (Thermo Fischer Scientific, Denmark) were placed in 6-well culture plates. One strain combination (ScottA and PL25) was selected based on the results of growth and *in vitro* virulence competition experiments. Two ml of ScottA culture were added to the upper chamber of the well and 2 ml of PL25 culture were added to the lower chamber (ensuring no contact between strains). Growth of strains in single cultures was also tested in separate wells in addition to growth of strains in direct contact (mixed in 1:1 ratio as described for growth experiments). The effect of cultivating the cells in the upper chamber in comparison to the lower chamber was also tested. For the growth experiments, cultures were incubated at 10°C for 10 days. Sampling was performed at day 0, 1 3, 5, 7 and 10. Each experiment was performed four independent times in duplicate.

For the *in vitro* virulence assay, individual or mixed *L*. *monocytogenes* strains were incubated at an initial cell density of 10^6^ CFU/ml at 10°C for 24 h (in 4ml TSB-Y in 6-well tissue culture plates). The i*n vitro* virulence assay was performed with i) single cultures, ii) mixed-strain culture (strains in contact during growth and infection assay), iii) co-culture without contact (strains grown together separated by the membrane) but in contact during virulence assay and iv) co-culture without contact (strains grown together separated by the membrane) and used individually for the virulence assay. The experiment was performed four independent times in triplicate.

To confirm that bacteria did not pass through the filter only one chamber was filled with bacterial culture and the media, incubated at 10°C for 10 days and CFU/ml of both chambers were determined.

### Statistical analysis

Data analysis was performed using Microsoft Excel® 2007 and SPSS 22.0 for Mac (SPSS Inc., Chicago, IL, United States). Differences in log CFU/ml at different time points, invasion efficiency and intracellular growth between single and mixed cultures were determined using independent t-test. To compare the mean values of multiple groups (contact/non-contact) we used Tukey’s HSD test. All experiments were performed at least three different times in duplicate for growth determination and in triplicate for virulence assays. Differences were considered to be significant for *P*-values <0.05.

## Results

### Characteristics of *L*. *monocytogenes* strains

We used four strains in this study: the human reference strain ScottA (4b, ST290), C5 (ST2) an isolate from dairy farm environment [36,37], the meat isolate PL25 (ST59 [38] and the cheese isolate 6179, which persisted in a food environment for at least 7 years (ST121) and harbors a truncated *inlA* resulting in attenuated invasion in Caco2 cells [39,40] (Table 1). Artificial antibiotic resistance against streptomycin or rifampicin could be introduced in these four *L*. *monocytogenes* strains to allow selective enumeration, thus resulting in 5 strains (both streptomycin and rifampicin resistance was introduced into strain ScottA; S1 Table). No significant difference in growth rates was observed between all antibiotic resistant *L*. *monocytogenes* strains. All strains reached a final cell density of 9 log CFU/ml within 10 days of incubation in TSB-Y at 10°C (S1 Fig). Additionally, the growth rate was equal between antibiotic sensitive and resistant strains.

### Growth competition between *L*. *monocytogenes* strains

**Fig 1:**
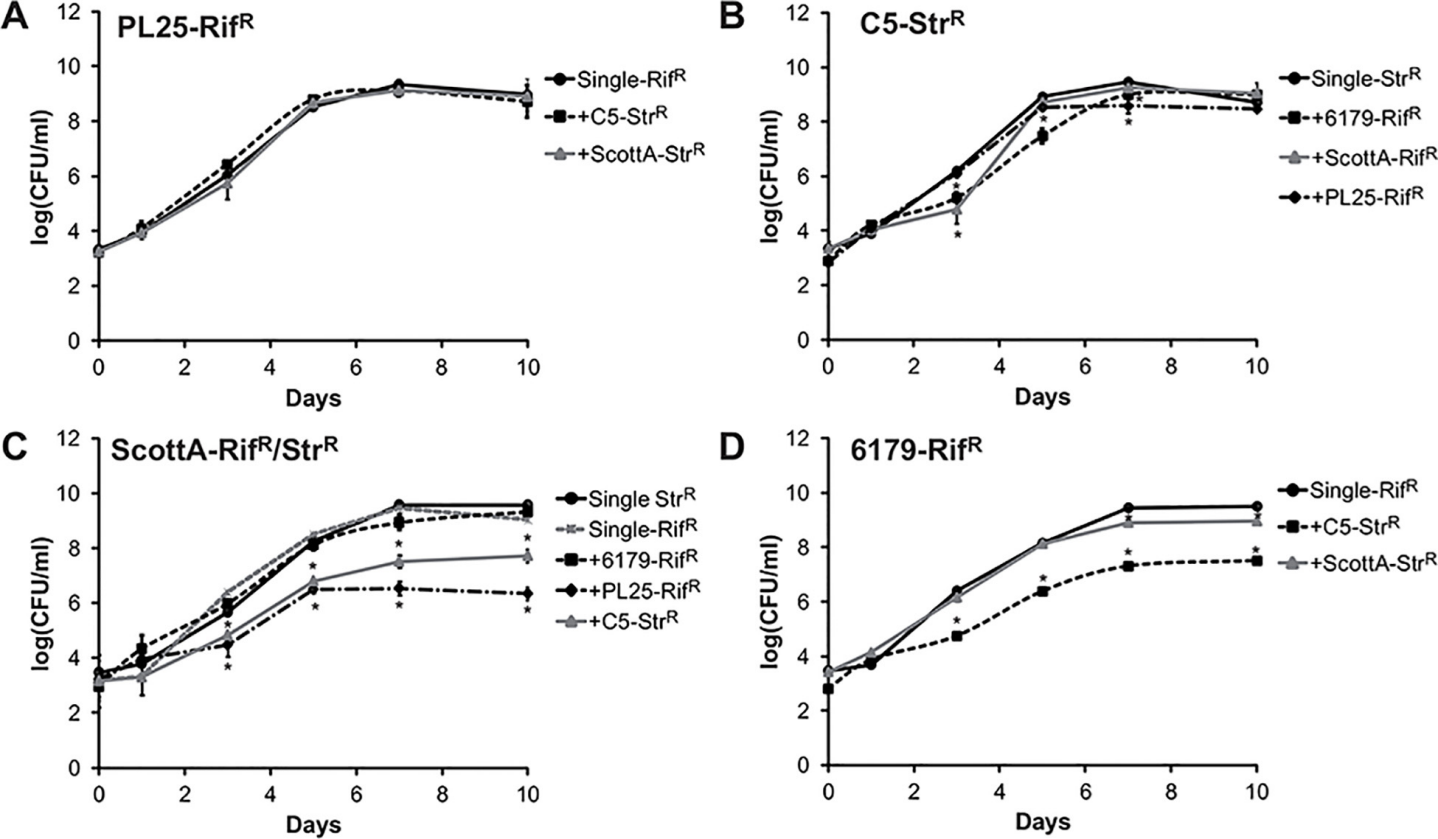
Growth competition of *L*. *monocytogenes* strains. *L*. *monocytogenes* strains (A) PL25-Rif^R^, (B) C5-Str^R^, (C) ScottA-Str^R^/Rif^R^ and (D) 6179-Rif^R^ were grown alone (single) and in the presence of a second *L*. *monocytogenes* strain in TSB-Y for 10 days at 10°C. Cultures were sampled on day 0, 1, 3, 5, 7 and 10; and CFUs were determined by plating serial dilutions on TSA-Y and TSA-Y supplemented with rifampicin or streptomycin. Data represented as log (CFU/ml) are mean values ± standard deviation of three biological replicates performed in duplicate. *indicate statistically significant differences between the co-culture and the corresponding single culture (P<0.05).

We compared the growth of each *L*. *monocytogenes* strain grown singly to that of the same strain grown in the presence of a second strain (in total 5 combinations) using a nutrient-rich media (TSB-Y) at 10°C for 10 days (Fig 1). In 3 of 5 strain combinations we observed a strong reduction of the growth kinetics when strains were grown in mixed culture compared to the single culture resulting in lower bacterial numbers at day 10; either of one strain or both strains (Fig 1).

Co-cultivation with strains C5 and PL25 decreased strain ScottA growth, resulting in lower 10 day populations (Fig 1C); whereas growth of C5 and PL25 was not affected by ScottA. In contrast co-cultivation of strain C5 and strain 6179 decreased the growth rate of both strains; however, growth of C5 was only slightly attenuated in the logarithmic growth phase, but reached equal cell density compared to single culture after 10 days. Growth of strain C5 was additionally reduced only at day 3 in the presence of ScottA. Furthermore, we detected lower population of strain 6179 in the presence of ScottA at day 7 and 10. Notably, in all combinations the growth of strain PL25 was never inhibited by the presence of other strains (Fig 1A).

Taken together we identified PL25 and C5 as strong competitor strains, whose growth was not (or only slightly) influenced by other strains resulting in all combinations in equal final cell density and two weak competitor strains, ScottA and 6179, which were overgrown by other strains.

### *In vitro* virulence of *L*. *monocytogenes* strains

**Fig 2:**
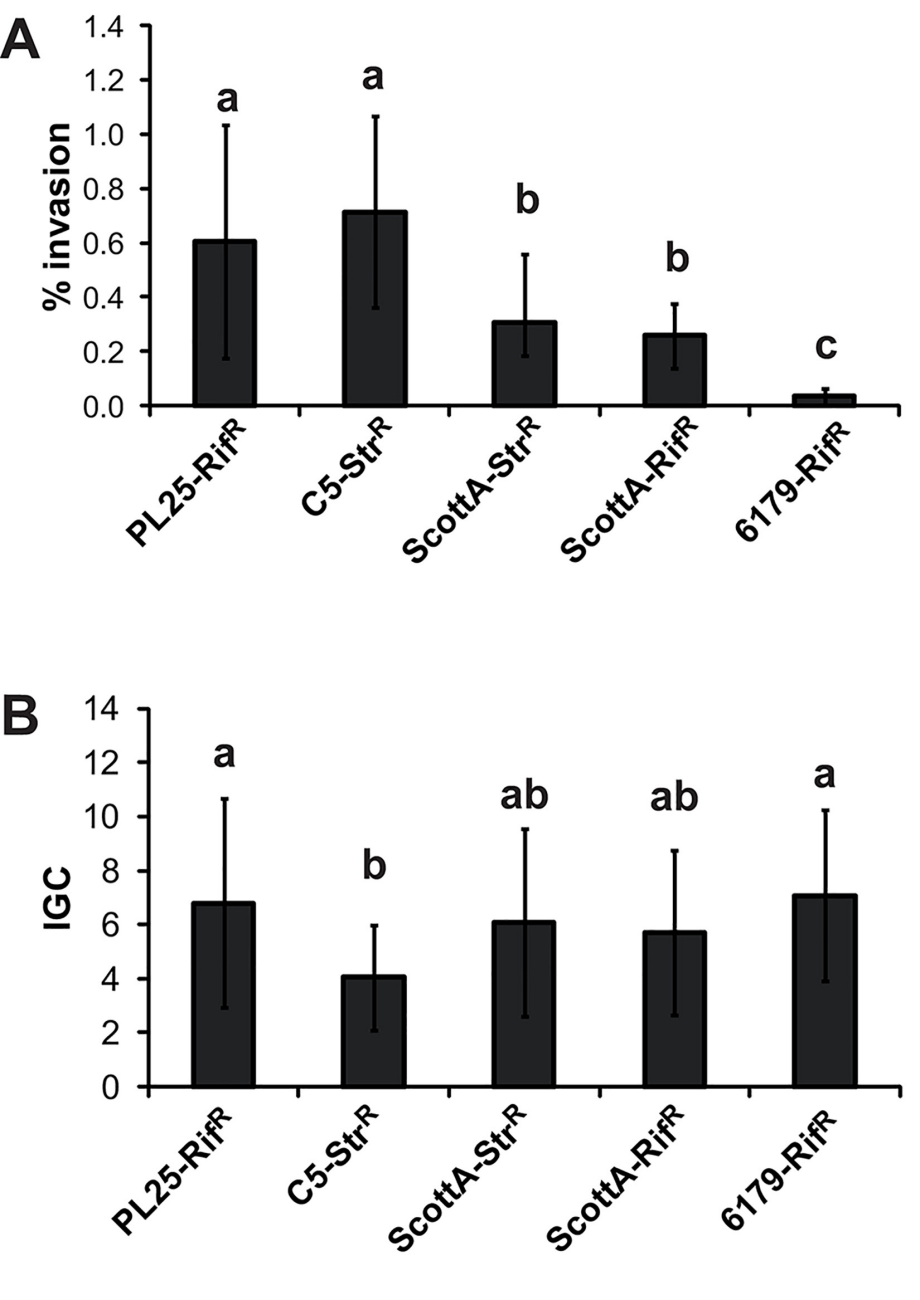
*In vitro* virulence potential of *L*. *monocytogenes* strains. (A)Invasion efficiency and (B) intracellular growth (IGC) of *L*. *monocytogenes* strains PL25-Rif^R^, C5-Str^R^, ScottA(Str^R^/Rif^R^) and 6179-Rif^R^ were determined using Caco2 cells. Bacteria were incubated for 1 day at 10°C in TSB-Y. Caco2 cells were infected for 1h with bacteria (multiplicity of infection of 25), incubated for 45 min (invasion) and 4h (intracellular growth) with gentamycin. IGC was calculated as the number of intracellular bacteria after 4h minus the number of bacteria after 45 min divided by the number of bacteria after 45min. Data, represented as % of invasion and IGC, are mean values ± standard deviation of three biological replicates performed in triplicate. Different letters indicate statistically significant differences between the strains (P<0.05). p-values are shown in S2 Table.

To test whether the fitness competition is associated with the virulence potential we determined the invasion efficiency and intracellular growth coefficient (IGC) of the single *L*. *monocytogenes* strains using human epithelial Caco2 cells (Fig 2). C5 and PL25, the strong competitors during growth, showed the highest invasion efficiency followed by ScottA (ranked among the tested strains as moderate invasive strain), and strain 6179 (ranked as a low invasive strain), which were both weak competitors during growth (Fig 2A). The differences in intracellular growth between the four strains were lower compared to the invasion efficiency: IGCs of strains 6179 and PL25 were only slightly but significantly higher compared to C5 (Fig 2B).

Our data suggest that the strains showing high invasiveness are stronger growth competitors compared to the modest or low invasive strains. Regarding intracellular growth, we could not detect any pattern.

### *In vitro* virulence competition between *L*. *monocytogenes* strains

We investigated whether the *in vitro* virulence potential of single *L*. *monocytogenes* strains affects the outcome of virulence competition using Caco2 cells.

**Fig 3:**
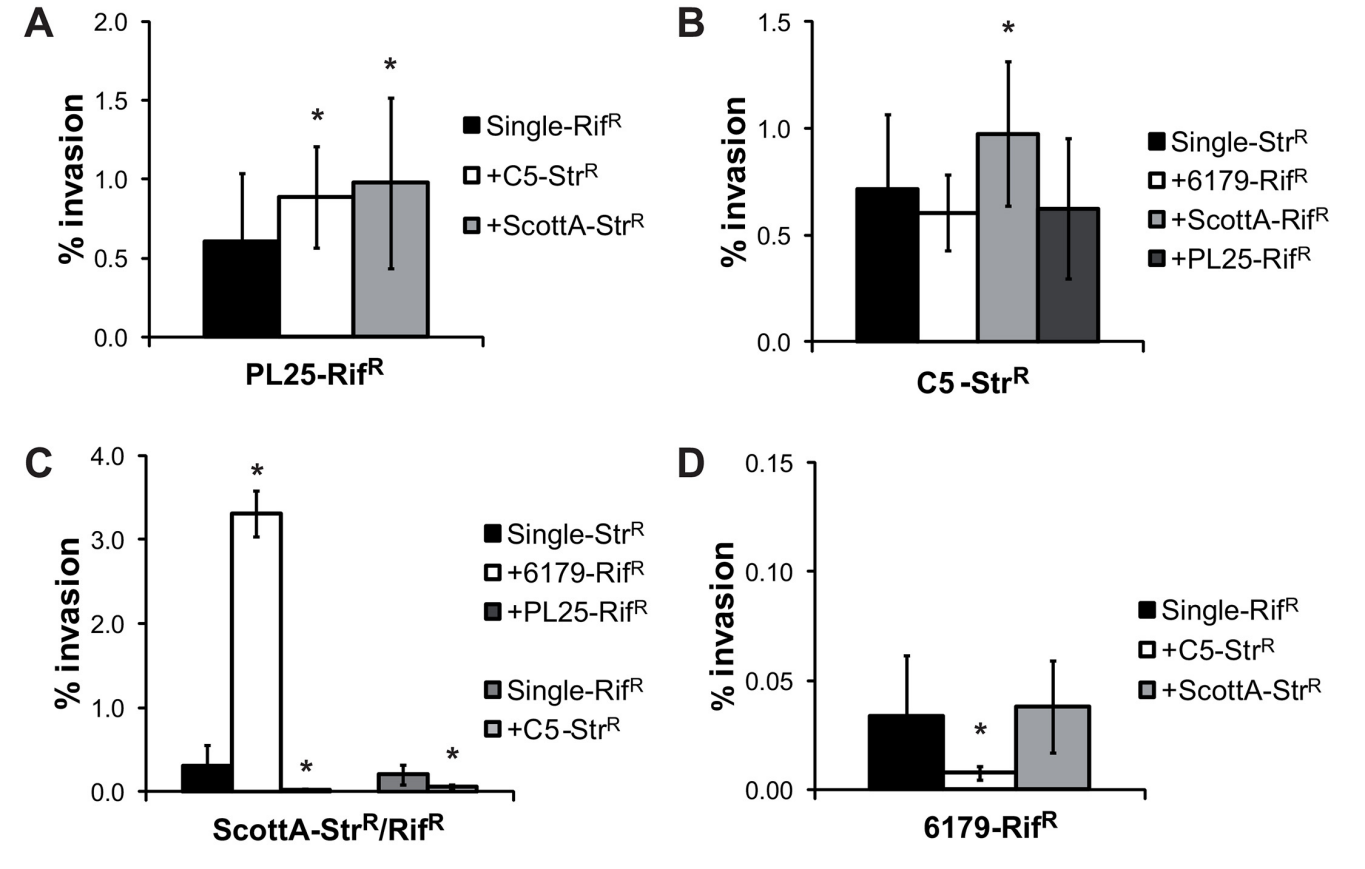
Effect of strain competition on the invasion efficiency of *L*. *monocytogenes* strains. Invasion efficiency (%) of *L*. *monocytogenes* strains (A) PL25-Rif^R^, (B) C5-Str^R^, (C) ScottA-Str^R^/Rif^R^ and (D) 6179-Rif^R^ grown alone (single) or in the presence of a second *L*. *monocytogenes* strain (1 day, 10°C, TSB-Y) was determined using Caco2 cells. Cells were infected for 1h with bacteria (multiplicity of infection of 25), and incubated for 45min (invasion) with gentamycin. Data, represented as % of invasion, are mean values ± standard deviation of three biological replicates performed in triplicate. *indicates significant difference of the mixed culture compared to the corresponding single culture (P<0.05). p-values are shown in S3 Table.

Invasion efficiency of the high invasive strains C5 and PL25 increased slightly, but significantly when co-cultured with the moderate invasive strain ScottA (Fig 3A and 3B). Strain PL25, the strongest growth competitor, also showed increased invasion efficiency in the presence of C5 (Fig 3A). However, these effects were only mosdest. Strain ScottA demonstrated attenuated invasion efficiency, when co-cultured with C5 or PL25 (Fig 3C), whereas its ability to invade into Caco2 cells increased in the presence of the low invasive strain 6179 up to 10-fold. Furthermore, the invasion efficiency of strain 6179 was significantly decreased when co-cultured with C5 (Fig 3D). These results show an invasion advantage for the higher invasive strain in several strain combinations, which can also be disadvantageous for the low invasive strains.

**Fig 4:**
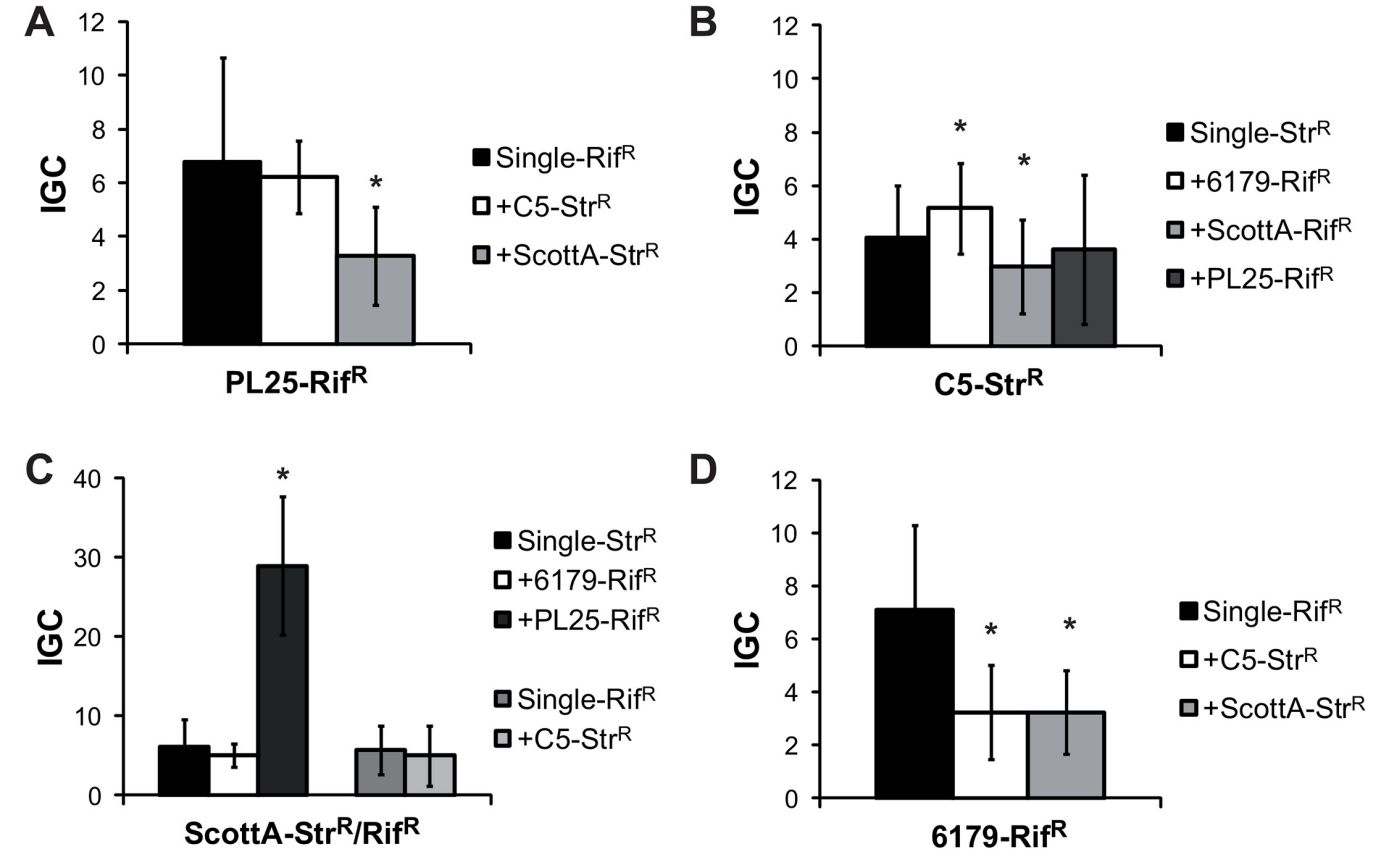
Effect of strain competition on the intracellular growth of *L*. *monocytogenes* strains in Caco2 cells. Intracellular growth (calculated as IGC) of *L*. *monocytogenes* strains (A) PL25-Rif^R^, (B) C5-Str^R^, (C) ScottA-Str^R^/Rif^R^ and (D) 6179-Rif^R^ grown alone (single) or in the presence of a second *L*. *monocytogenes* strain (1 day, 10°C, TSB-Y) was determined using Caco2 cells. Caco2 cells were infected for 1h with bacteria (multiplicity of infection of 25), and incubated for 4h (intracellular growth) with gentamycin. IGC was calculated as the number of intracellular bacteria after 4h minus the number of bacteria after 45min divided by the number of bacteria after 45min. Data, represented as IGC, are mean values ± standard deviation of three biological replicates performed in triplicate. *indicates significant difference of the mixed culture compared to the corresponding single culture (P<0.05). P-values are shown in S3 Table.

Strain-competition also affected intracellular growth (measured as IGC) in the Caco2 cells, resulting in a complex pattern (Fig 4). In contrary to the invasion efficiency the IGC of strain PL25 and C5 was significantly lower in the presence of ScottA (Fig 4A and 4B). Reciprocally, the intracellular growth of strain ScottA was 30-fold increased in the presence of PL25 (Fig 4C), whereas invasion efficiency was decreased (15-fold reduction) resulting in an overall higher number of intracellular bacteria after 4 hours. Interestingly, the ICG of strain 6179, the invasion-attenuated strain, was reduced in the presence of other strains (Fig 4D), indicating that the overall *in vitro* virulence potential of 6179 (including both invasion and intracellular growth) was reduced in the presence of other strains.

### Contact-dependent growth and *in vitro* virulence competition of *L*. *monocytogenes*

We tested whether the effect of strain competition on growth and *in vitro* virulence was contact-dependent using strains ScottA and PL25. We selected this strain combination because we observed high differences in growth (for strain ScottA), invasion and intracellular growth (for both strains) due to co-cultivation. Strains were separated by a 0.4 μm PET membrane, which allows the exchange of produced molecules but does not allow the two strains to inter-mix.

**Fig 5:**
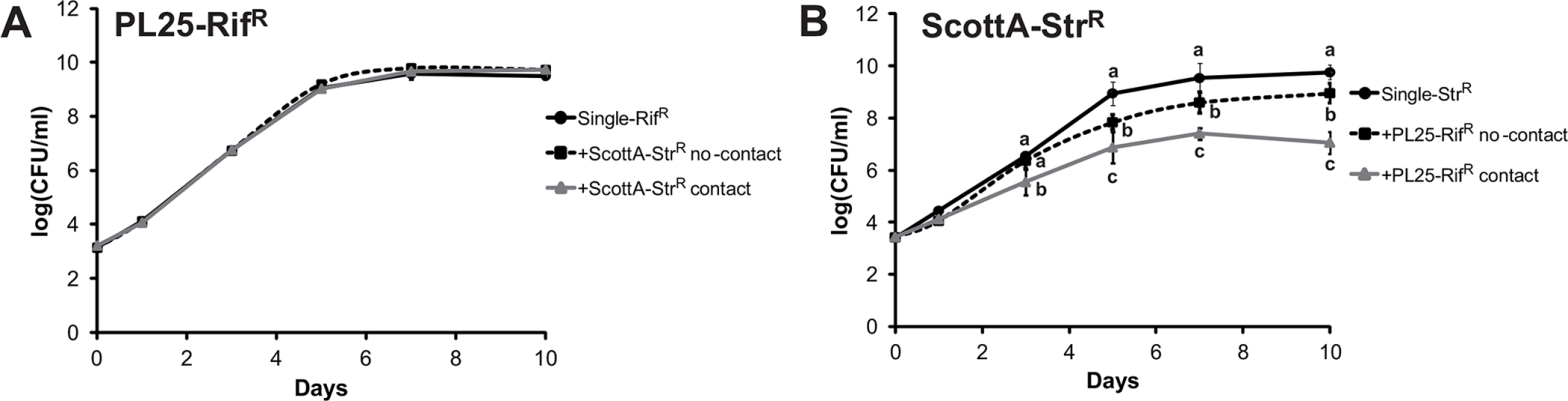
Cell-contact dependent growth competition of *L*. *monocytogenes* strains. *L*. *monocytogenes* strains (A) PL25-Rif^R^ and (B) ScottA-Str^R^ were grown alone (single), mixed (contact) and in the presence of the second *L*. *monocytogenes* strain separated by a 0.4 μm membrane (no-contact) in TSB-Y for 10 days at 10°C. Data represented as log (CFU/ml) are mean values ± standard deviation of three biological replicates performed in duplicate. Different letters indicate statistically significant differences between single culture, contact and non-contact co-cultivation at the different time points (P<0.05).

Growth of strain ScottA was significantly reduced in the presence of strain PL25 separated by a membrane at day 5, 7 and 10 compared to the single strain (Fig 5B). However, growth reduction was significantly higher when cell contact between the two strains was possible (log CFU/ml reduction of 2–2.7 versus 0.8–1.1).

**Fig 6:**
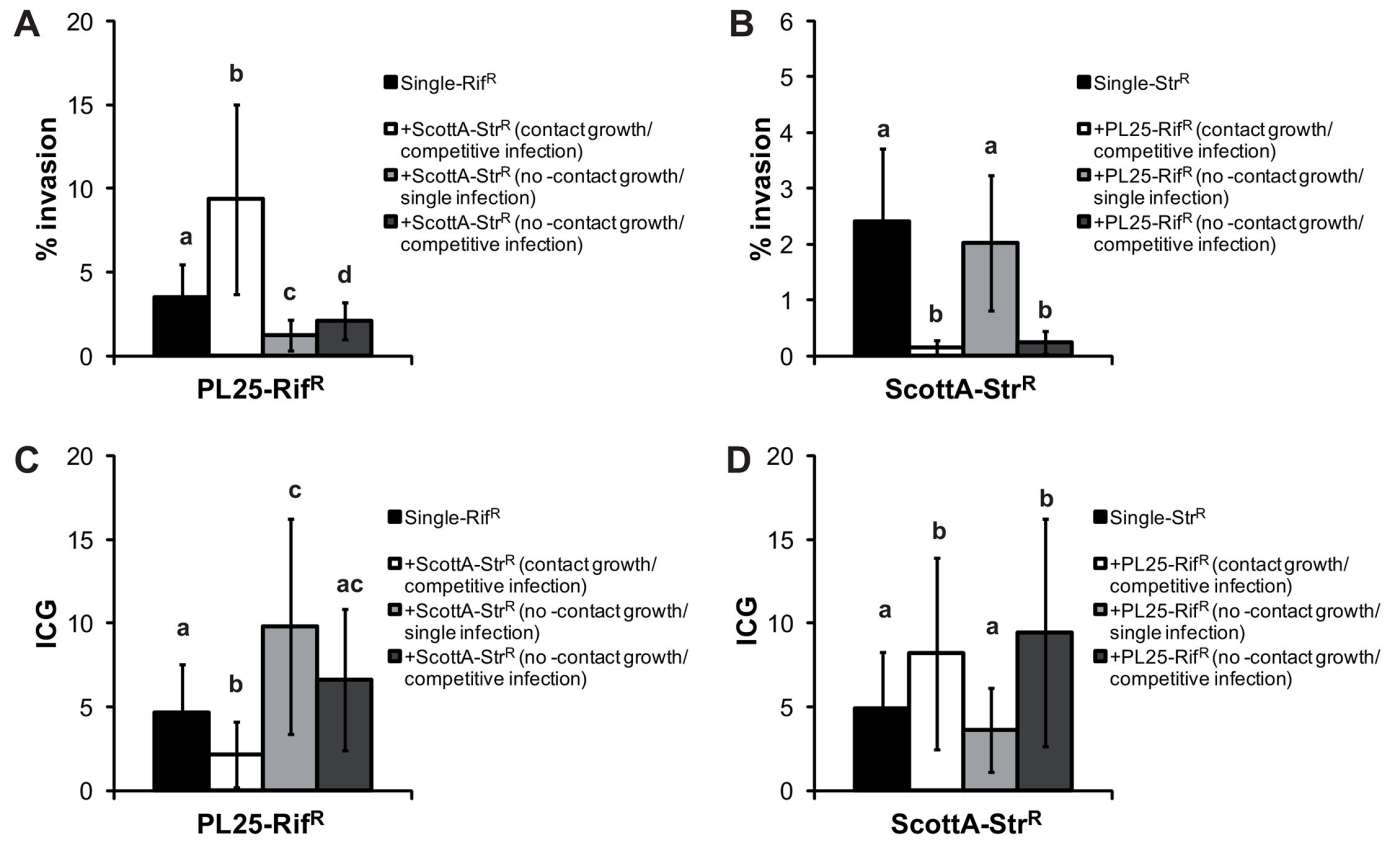
Cell-contact-dependent virulence competition of *L*. *monocytogenes* strains. (A&B) Invasion efficiency (%) and (C&D) intracellular growth (IGC) were determined for PL25-Rif^R^ and ScottA-Str^R^ using i) single cultures, ii) mixed culture (strains in contact during growth and infection assay), iii) co-culture without cell-contact (strains grown together separated by the membrane) and used singly for the virulence assay and iv) co-culture without cell-contact (strains grown together separated by the membrane), and in contact during virulence assay. Data, represented as % of invasion and IGC, are mean values ± standard deviation of three biological replicates performed in triplicate. Different letters indicate statistically significant differences between the conditions (P<0.05). P-values are shown in S4 Table.

To investigate the effect of cell-contact on *in vitro* virulence, we incubated the strains ScottA and PL25 at 10°C for 1 day either individually or mixed and performed both single-strain and competitive infection. Notable, the level of invasion in this experiment was higher compared to Fig 3. The reason might be the different culture volumes and reservoirs used in these experiments. But the observed difference between single and mixed culture were equal. The results indicate a complex pattern (Fig 6). Increased invasion efficiency of PL25 in the presence of Scott A was only observed when cell-contact growth was possible prior to infection (Fig 6A). Intracellular growth of strain PL25 decreased only if contact with strain ScottA was possible. We observed even an increased IGC of strain PL25 when the strains were co-cultivated without contact and infected alone.

In contrast, reduced invasion efficiency (Fig 6B) and increased intracellular growth (Fig 6D) was only observed for strain Scott A together with PL25, regardless of whether cell contact prior to infection was possible or not. Our data suggest that competition for entry and replication into Caco-2 cells occurs at two different levels: i) during co-cultivation prior to infection and ii) during the infection process.

## Discussion

Our study demonstrates that fitness competition occurs between different *L*. *monocytogenes* strains and that strong growth competitors, whose growth was not or only slightly attenuated by other strains, showed high invasiveness compared to weak fitness competitors. Since the strains displayed equivalent growth kinetics in single cultures, we can exclude that the observed differences are due to distinct growth potential of the strains. This was also pointed out in a recent study showing that fitness differences between *L*. *monocytogenes* strains during the enrichment procedure were due to strain competition [30].

In our study we used four genetically distinct strains: two serotype 4b strains (lineage I, ST2 and ST290), one 1/2a strain (lineage 2, ST121) and one ST59 strain belonging to serogroup 1/2b (lineage I, ST59). We did not observe the dominance of any specific lineage or serotype. Obviously, to test whether strains of certain lineages and serotypes have advantages in strain competition a higher number of strain combinations might be necessary. Furthermore, more detailed characteristics such as sequence type, stress response or virulence potential should be considered as factors influencing strain competition. In relation to that, Gorski et al. [30] investigated *L*. *monocytogenes* strain competition of 4b and 1/2a strains during enrichment. The authors demonstrated that the observed differences in strain fitness did not correlate with serotype or the genetic lineages. Additionally, Daily et al. [41] reported that competition during selective enrichment between non-pathogenic foodborne bacteria and *L*. *monoyctogenes* was not associated with any specific serotype of *L*. *monocytogenes*. In contrast, Bruhn et al. [31] reported that lineage 2 strains outcompeted lineage 1 strains in selective enrichments.

We found that the strong fitness competitors in a nutrient rich broth at 10°C show high invasion efficiency, suggesting a possible association between fitness outside the host and invasivness. There is evidence for a close link between fitness, stress response and pathogenicity in *L*. *monocytogenes*. The major virulence gene regulator PrfA is regulated by the transcription factor σ^B^, dominant in the general stress response [42]. Additionally, major virulence genes such as *inlA* and *inlB*, are coregulated by PrfA and σ^B^ [43]. The expression and activation level of PrfA seems to have an essential role in the balance between host and environmental survival skills in *L*. *monocytogenes* [44]. It has been shown that the constitutive expression of PrfA resulted in a hypervirulent, but low-fitness phenotype at 37°C [45]. In our study we cultivated the bacteria at 10°C; therefore, cold stress and adaptation might be one additional factor influencing the invasion and intracellular growth. Recently we were able to show that *L*. *monocytogenes* stored at 4°C in milk had higher invasiveness compared to storage at 25 and 30°C [35], suggesting a correlation between temperature and invasion. Of note, the lowest invasive strain 6179 harbors a truncated *inlA* gene, being the main factor for attenuated invasiveness into Caco2 cells [40]. Although reported to be able to persist for 7 years in a food-processing environment, strain 6179 is a weak fitness competitor under the tested conditions. However, the food-processing environment is different than that of a nutrient rich growth medium and cells are exposed to different stresses and nutrient availability.

Since different *L*. *monocytogenes* strains can be present in the same food, and infection with more than one strain can occur, we investigated the *in vitro* virulence competition of multiple strains of *L*. *monocytogenes*. In several listeriosis outbreaks more than one *L*. *monocytogenes* strain has been involved: for example, four different *L*. *monocytogenes* strains were associated with the recent cantaloupe listeriosis outbreak in the US [46,47]. In 2009/2010 two distinct serotype 1/2a strains were involved in a multinational outbreak traced back to a traditional Austrian Quargel cheese [48,49]; and two closely related strains were responsible for a large listeriosis outbreak in Canada in 2008 [50]. Furthermore, Tham et al. [51] documented a listeriosis patient being infected with two different *L*. *monocytogenes* strains.

Investigating the effect of co-cultivation on the *in vitro* virulence of two *L monocytogenes* strains in a nutrient rich media (mimicking the food environment) we showed that the high invasion potential results in an advantage in invasion competition. In certain combinations co-cultivation boosted in the invasion efficiency of the more invasive and could even attenuate that of the strain with the lower invasion. In all tested combinations the strain displaying higher invasion potential was never outcompeted by the lower invasive strain.

Regarding intracellular growth we could not detect any trend. Since individual intracellular growth of most strains were almost similar, the observed competition inside Caco-2 cells was rather strain-dependent and did neither correlate with intracellular growth potential nor with growth competition in nutrient broth. However the intracellular growth of the low virulent strain 6179, whose invasion potential was reduced by the presence of strain C5, was attenuated by co-cultivation with C5 and ScottA.

Of particular interest, is the competition between ScottA and PL25; invasion efficiency of ScottA (the strain with lower invasiveness) decreased, but intracellular growth increased when the strains were co-cultivated, whereas the effect on PL25 was opposite. Although growth of ScottA was suppressed by PL25 in a nutrient rich media, ScottA becomes a stronger competitor in the intracellular environment of Caco-2 cells. This underlines that the environments inside and outside of the infected host cell are different resulting in distinct metabolic responses of *L*. *monocytogenes* due to different carbon utilization [52]. There is a close link between carbon source utilization and regulation of PrfA, whose expression and activation might be responsible for the observed effect in competition between strain ScottA and PL25.

Our cell-contact dependent co-cultivation data suggest a role of cell-contact in growth inhibition, at least for strains ScottA and PL25. There is evidence that contact dependent inhibition (CDI) systems play an important role in bacterial competition mainly in Gram-negative bacteria [8,53] However, recent studies showed that Gram-positive bacteria harbor proteins with high sequence similarities to CDI proteins such as rearrangement hotspot (rhs) proteins [9,54]. Schmitz-Esser et al. [40] could show that *L*. *monocytogenes* strains of ST121 including strain 6179 harbor RHS proteins (whose function is unknown yet), suggesting a better competition of these strains against other bacteria in food producing environments. However, in our study we did not observe any fitness advantage of strain 6179 indicating that the effect of RHS proteins on growth inhibition might be restricted to other bacteria species or under other conditions.

Our data suggest that other factors like the Agr- or the autoinducer 2 LuxS system [55–58], shown to be involved in quorum sensing in *L*. *monocytogenes*, could have a minor influence in *L*. *monocytogenes* inter-strain growth inhibition. Further studies using a higher number of tested strain-combinations are required to elucidate the mechanism involved in inter-strain growth inhibition in *L*. *monocytogenes*.

Investigating the role of cell-contact on *in vitro* virulence competition revealed a complex pattern. Cell-contact prior to infection influenced only the behavior of PL25, whereas ScottA showed equal invasion and intracellular growth, both with and without prior cell contact. This was contradictory to growth experiments where cell-contact between the strains reduced the fitness of ScottA suggesting a different underlying mechanism. The invasiveness of ScottA was only attenuated when co-infected with the second strain, showing that *in vitro* virulence competition can take place at two different levels: before infection during cell-contact-dependent co-cultivation potentially inducing the expression of virulence factors and during the infection process competing for the entry into the host cell.

The expression of primary virulence factors of *L*. *monocytogenes* could be affected by co-cultivation of other bacteria including other *Listeria* species. Tan et al. [59] showed that virulence-related genes of *L*. *monocytogenes* have been significantly downregulated when co-cultured with *Bifidobacterium longum*. Direct strain competition during infection, has been described for probiotic bacteria and *L*. *monocytogenes*. Investigating the ability of probiotic bacteria to prevent adhesion and invasion of the pathogen in human intestinal mucus or Caco-2 cells revealed that the effect depends on the specific probiotic strain and the relative concentrations of the two bacteria [60–62].

In conclusion we showed that co-cultivation of *L*. *monocytogenes* strains can lead to differences in fitness, invasiveness and intracellular growth and demonstrated that cell contact plays a certain role in growth inhibition and partially in *in vitro* virulence competition. Our results show that the growth of *L*. *monocytogenes* can not only be inhibited by other species like *L*. *innocua*, but also by strains of the same species leading potentially to biased detectability during enrichment procedures. Additionally, the presence of more than one *L*. *monocytogenes* strain in one food product can increase the infection rate due to synergistic effects on the virulence potential.

## Supporting Information

S1 Fig Growth of *L*. *monocytogenes* strains used in this study. (DOCX)

S1 Table MICs of streptomycin and rifampicin of the parental and resistant *L*. *monocytogenes* strains. (DOCX)

S2 Table p-values for Fig 2 (Invasion and intracellular growth of *L*. *monocytogenes* strains). (DOCX)

S3 Table p-values (independent t-test) for Fig 3 (Effect of strain competition on the invasion efficiency of *L*. *monocytogenes* strains.). and Fig 4 (Effect of strain competition on the invasion efficiency of *L*. *monocytogenes* strains.). (DOCX)

S4 Table p-values (Tukey’s HSD test) for Fig 6. (Cell-contact-dependent virulence competition of *L*. *monocytogenes* strains.).
(DOCX)
