# Supplementary material for: Highly Invasive Listeria monocytogenes Strains Have Growth and Invasion Advantages in Strain Competition
Source: PLoS One. 2015 Nov 3;10(11):e0141617. doi: 10.1371/journal.pone.0141617 (PMC4631365; doi:10.1371/journal.pone.0141617)
Supplement: S1 Fig — (DOCX) [file pone.0141617.s001.docx]

**S1 Fig. Growth of *L. monocytogenes* strains used in this study.**

**
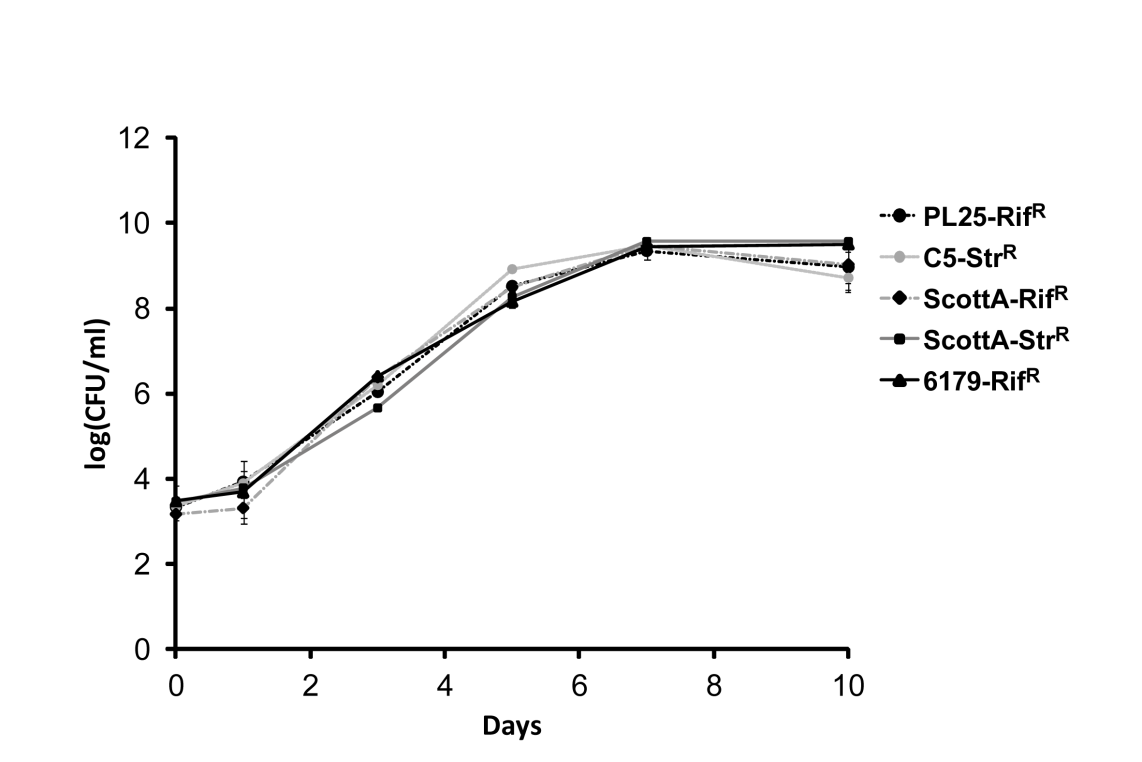
**

Growth dynamics of *L. monocytogenes* strains resistant to streptomycin (Str^R^) or rifampicin (Rif^R^) in TSB-Y for 10 days at 10°C. Cultures were sampled on day 0, 1, 3, 5, 7 and 10; and CFUs were determined by plating serial dilutions on TSA-Y and TSA-Y supplemented with rifampicin or streptomycin. Values, represented as log (CFU/ml), are mean values ± standard deviation of three biological replicates performed in duplicate.
