# Supplementary material for: Highly Invasive Listeria monocytogenes Strains Have Growth and Invasion Advantages in Strain Competition
Source: PLoS One. 2015 Nov 3;10(11):e0141617. doi: 10.1371/journal.pone.0141617 (PMC4631365; doi:10.1371/journal.pone.0141617)
Supplement: S1 Table — (DOCX) [file pone.0141617.s002.docx]

**S1 Table. MICs of streptomycin and rifampicin of the parental and resistant *L. monocytogenes* strains.**

| Strain | Antibiotics | MIC (µg/ml)  parental strain | MIC (µg/ml)  resistant strain |
| --- | --- | --- | --- |
| C5 | streptomycin | 100 | 2000 |
| ScottA | rifampicin | <0.31 | >800 |
| ScottA | streptomycin | 100 | 4000 |
| 6179 | rifampicin | <0.31 | >800 |
| PL25 | rifampicin | <0.31 | 800 |
