# Supplementary material for: Highly Invasive Listeria monocytogenes Strains Have Growth and Invasion Advantages in Strain Competition
Source: PLoS One. 2015 Nov 3;10(11):e0141617. doi: 10.1371/journal.pone.0141617 (PMC4631365; doi:10.1371/journal.pone.0141617)
Supplement: S2 Table — (DOCX) [file pone.0141617.s003.docx]

**S2 Table: p-values for Fig. 2** (Invasion and intracellular growth of *L. monocytogenes* strains)

| **p-values* (Fig2A)** | PL25-Rif^R^ | C5-Str^R^ | ScottA-Str | ScottA-Rif^R^ | 6179-Rif^R^ |
| --- | --- | --- | --- | --- | --- |
| PL25-Rif^R^ | 1.000 |  |  |  |  |
| C5-Str^R^ | 0.321 | 1.000 |  |  |  |
| ScottA-Str^R^ | <0.001 | <0.001 | 1.000 |  |  |
| ScottA-Rif^R^ | <0.001 | <0.001 | 0.976 | 1.000 |  |
| 6179-Rif^R^ | <0.001 | <0.001 | <0.001 | <0.001 | 1.000 |

| **p-values* (Fig2B)** | PL25-Rif^R^ | C5-Str^R^ | ScottA-Str | ScottA-Rif^R^ | 6179-Rif^R^ |
| --- | --- | --- | --- | --- | --- |
| PL25-Rif^R^ | 1.000 |  |  |  |  |
| C5-Str^R^ | <0.001 | 1.000 |  |  |  |
| ScottA-Str^R^ | 0.979 | 0.053 | 1.000 |  |  |
| ScottA-Rif^R^ | 0.770 | 0153 | 0.999 | 1.000 |  |
| 6179-Rif^R^ | 1.000 | <0.001 | 0.843 | 0.421 | 1.000 |

***** p-values (Tukey’s HSD test) were calculated between the mean values (invasion-Fig2A and intracellular growth (ICG)- Fig2B) of *L. monocytogenes* strains PL25-Rif^R^, C5-Str^R^, ScottA(Str^R^/Rif^R^) and 6179-Rif^R^
