# Supplementary material for: Highly Invasive Listeria monocytogenes Strains Have Growth and Invasion Advantages in Strain Competition
Source: PLoS One. 2015 Nov 3;10(11):e0141617. doi: 10.1371/journal.pone.0141617 (PMC4631365; doi:10.1371/journal.pone.0141617)
Supplement: S3 Table — (DOCX) [file pone.0141617.s004.docx]

**S3 Table: p-values (independent t-test) for Fig.3** (Effect of strain competition on the invasion efficiency of *L. monocytogenes* strains.) **and Fig. 4** (Effect of strain competition on the invasion efficiency of *L. monocytogenes* strains.)

| **Single culture** | **Combination** | **p-value^*^** | |
| --- | --- | --- | --- |
|  |  | **Invasion** | **IGC** |
| PL25-Rif^R^ | PL25-Rif^R^ +C5-Str^R^ | 0.030 | 0.670 |
| PL25-Rif^R^ | PL25-Rif^R^ +ScottA-Str^R^ | <0.001 | <0.001 |
| C5-Str^R^ | C5-Str^R^ +6179-Rif^R^ | 0.178 | 0.015 |
| C5-Str^R^ | C5-Str^R^ +ScottA-Rif^R^ | 0.010 | 0.041 |
| C5-Str^R^ | C5-Str^R^ +PL25-Rif^R^ | 0.830 | 0.403 |
| ScottA-Str^R^ | ScottA-Str^R^ +6179-Rif^R^ | <0.001 | 0.313 |
| ScottA-Str^R^ | ScottA-Str^R^ +PL25-Rif^R^ | <0.001 | <0.001 |
| ScottA-Rif^R^ | ScottA-Rif^R^ +C5**-**Str^R^ | <0.001 | 0.759 |
| 6179-Rif^R^ | 6179-Rif^R^ +C5**-**Str^R^ | <0.001 | <0.001 |
| 6179-Rif^R^ | 6179-Rif^R^ +ScottA-Str^R^ | 0.515 | <0.001 |

* p-values (independent t-test) were calculated between the mean values (invasion-Fig.3. and intracellular growth (ICG)-Fig.5.) of the co-culture and the corresponding single culture of *L. monocytogenes* strains (A) PL25-Rif^R^, (B) C5-Str^R^, (C) ScottA-Str^R^/Rif^R^ and (D) 6179-Rif^R^ grown alone (single) or in the presence of a second *L. monocytogenes* strain.
