# Supplementary material for: Highly Invasive Listeria monocytogenes Strains Have Growth and Invasion Advantages in Strain Competition
Source: PLoS One. 2015 Nov 3;10(11):e0141617. doi: 10.1371/journal.pone.0141617 (PMC4631365; doi:10.1371/journal.pone.0141617)
Supplement: S4 Table — (Cell-contact-dependent virulence competition of L. monocytogenes strains.). (DOCX) [file pone.0141617.s005.docx]

**S4 Table: p-values (Tukey’s HSD test) for Fig. 6.** (Cell-contact-dependent virulence competition of *L. monocytogenes* strains.)

| **Condition 1** | **Condition 2** | **P value** | |
| --- | --- | --- | --- |
|  |  | **Invasion** | **IGC** |
| Single PL25-Rif^R^ | PL25-Rif^R^+ScottA-Str^R^ (contact growth/competitive infection) | <0.001 | 0.001 |
| Single PL25-Rif^R^ | PL25-Rif^R^+ScottA-Str^R^ (no contact growth/single infection) | <0.001 | 0.003 |
| Single PL25-Rif^R^ | PL25-Rif^R^+ScottA-Str^R^ (no contact growth/competitive infection) | 0.003 | 0.107 |
| PL25-Rif^R^+ScottA-Str^R^ (contact growth/competitive infection) | PL25-Rif^R^+ScottA-Str^R^ (no contact growth/single infection) | <0.001 | <0.001 |
| PL25-Rif^R^+ScottA-Str^R^ (contact growth/competitive infection) | PL25-Rif^R^+ScottA-Str^R^ (no contact growth/competitive infection) | <0.001 | <0.001 |
| PL25-Rif^R^+ScottA-Str^R^ (no contact growth/single infection) | PL25-Rif^R^+ScottA-Str (no contact growth/competitive infection) | 0.002 | 0.053 |
| Single ScottA-Str^R^ | ScottA-Str^R^+PL25-Rif^R^ (contact growth/competitive infection) | <0.001 | 0.019 |
| Single ScottA-Str^R^ | ScottA-Str^R^+PL25-Rif^R^ (no contact growth/single infection) | 0.292 | 0.090 |
| Single ScottA-Str^R^ | ScottA-Str^R^+PL25-Rif^R^ (no contact growth/competitive infection) | <0.001 | 0.006 |
| ScottA-Str^R^+PL25-Rif^R^ (contact growth/competitive infection) | ScottA-Str^R^+PL25-Rif^R^ (no contact growth/single infection) | <0.001 | <0.001 |
| ScottA-Str^R^+PL25-Rif^R^ (contact growth/competitive infection) | ScottA-Str^R^+PL25-Rif^R^ (no contact growth/competitive infection) | 0.088 | 0.493 |
| ScottA-Str^R^+PL25-Rif^R^ (no contact growth/single infection) | ScottA-Str^R^+PL25-Rif^R^ (no contact growth/competitive infection) | <0.001 | <0.001 |

*p-values (Tukey’s HSD test) were calculated between the mean values (invasion-Fig.6A and B and intracellular growth (ICG)-Fig.6 C and D) for strains PL25-Rif^R^ and ScottA-Str^R^ using i) single cultures, ii) mixed culture (strains in contact during growth and infection assay), iii) co-culture without cell-contact (strains grown together separated by the membrane) and used singly for the virulence assay and iv) co-culture without cell-contact (strains grown together separated by the membrane), and in contact during virulence assay.
